# The Mussels That Came in From the Cold: Long‐Term Effects of the Population Collapse in the 1960s May Explain Low Abundances of Boreal Mussels in the Subarctic Despite the Warming

**DOI:** 10.1002/ece3.73763

**Published:** 2026-06-03

**Authors:** Julia Marchenko, Vadim Khaitov, Larisa Basova, Evgeny Genelt‐Yanovskiy, Sergey Malavenda, Petr Strelkov

**Affiliations:** ^1^ Faculty of Biology St. Petersburg State University St. Petersburg Russia; ^2^ Kandalaksha State Nature Reserve Kandalaksha Russia; ^3^ Algology Laboratory Murmansk Marine Biological Institute of the Russian Academy of Sciences Murmansk Russia; ^4^ Department of Earth and Environmental Sciences University of Exeter, Penryn Campus Penryn UK; ^5^ Natural Science and Technologies Institute Murmansk Arctic University Murmansk Russia

**Keywords:** Allee effect, Arctic warming, demography, long‐term population dynamics, *Mytilus edulis*, *Mytilus trossulus*

## Abstract

Boreal species are generally stressed by climate warming at the southern limit of their distribution and are expected to thrive at the poleward limit. Blue mussels were the heralds of climate warming in the Arctic, appearing in Spitsbergen a quarter of a century ago after the absence of a millennium. Here we tested the hypothesis that warming has a positive effect on subarctic mussels, too. We gathered and analyzed two datasets from the Murman Coast (Russia), 1000 km southeast of Spitsbergen across the Barents Sea. The first dataset contained data on the age structure of a mussel population monitored almost annually in 2001–2020, while the second dataset contained all comparable historical data on mussel biomass: 173 estimates of mean biomass at 62 sites along the entire coast collected in 1933–2020. Analysis of the first dataset showed that temperature had a positive effect on recruitment and survival. However, no positive effect was found in the analysis of the long‐term dynamics. The abundance of mussels was high until the 1960s and then dropped tenfold. The collapse coincided with the climate cooling. The populations partially recovered in the subsequent unprecedentedly warm period, but no direct relationship between the recovery and temperature was found. We speculate that the subarctic mussels could not fully recover due to long‐term consequences of the collapse. The Allee effect in their depleted populations might have limited recovery for decades. Besides, the fitness of blue mussel populations is currently undermined by competition and hybridization between the two cryptic species, the native *Mytilus edulis* and the invasive *M. trossulus* , which presumably naturalized after the collapse.

## Graphical abstract

Arctic warming is predicted to have a positive effect on boreal species. To test this hypothesis, we gathered and analyzed two datasets on blue mussels *Mytilus* from the Murman Coast of the Barents Sea. The first dataset contained data on the age structure of a mussel population monitored from 2001 to 2020, while the second dataset contained data on mussel biomass from 1933 to 2020. Analysis of the first dataset showed that temperature had a positive effect on recruitment and survival. However, no positive effect was found in the analysis of the long‐term dynamics.

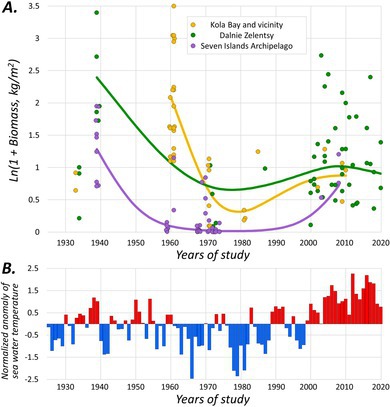

## 1 Introduction

While boreal species are generally stressed by climate warming at the southern limit of their distribution, they are expected to thrive at the poleward limit, where their abundances increase and ranges expand (Parmesan 2006; Poloczanska et al. 2016). In the Arctic Ocean these processes are part of the ecosystem regime shift referred to as Atlantification and borealization (Fossheim et al. 2015; Polyakov et al. 2020; Csapó et al. 2021; Ingvaldsen et al. 2021). The changes in pan‐Arctic marine macrophyte communities are a good example: the analysis of long‐term data series on brown algae and eelgrass indicates both an increase in vegetation and an expansion of distribution ranges northwards since the 20th century (Krause‐Jensen et al. 2020). Boreal blue mussels of the genus *Mytilus* are considered as beneficiaries of climate warming and the heralds of borealization in the Arctic (Berge et al. 2005; Leopold et al. 2019; Csapó et al. 2021).

Two species of blue mussels, *M. edulis* and *M. trossulus* , are ubiquitous in boreal and subarctic seas (Gosling 2021). Being cryptic species, they are usually not distinguished in ecological studies (Katolikova et al. 2016). Blue mussels are powerful ecosystem engineers in coastal ecosystems and important aquaculture species (Seed and Suchanek 1992; www.fao.org). Mussels are also significant in stratigraphical respect. The presence of subfossil *Mytilus* shells in the Quaternary sediments is a reliable marker of boreal conditions in the Arctic, as shown for the Kara Sea (Troitsky 1966), Northern Greenland (Dyke et al. 1996) and Novaya Zemlya (Mangerud et al. 2008). In Spitsbergen blue mussels appeared and disappeared throughout the Late Pleistocene–Holocene in accordance with climate fluctuations (Blake 2006; Hansen et al. 2011; Mangerud and Svendsen 2018). The current warming was marked by a new invasion in the early aughts (Berge et al. 2005), so that the researchers could observe this striking event just as it was unfolding.

Taking into account the evidence from Spitsbergen, it can be assumed that mussel abundance should be positively correlated with water temperature not only in the high Arctic but also in the low Arctic (or subarctic according to Dunbar 1953), for example, in the southern Barents Sea, where mussels, as far as it is known, have always been present (Zenkevich 1963). It also stands to reason that these dynamics should be evident on time scales shorter than millennia. Unfortunately, these assumptions cannot be reliably tested due to the paucity of data on subarctic mussels. To our knowledge, only two studies, both based on limited data, have addressed mussel dynamics on the decadal time scale at these latitudes (Feder et al. 2003; Marchenko et al. 2023), and no obvious link between mussel abundance and water temperature has been found in either of them. In contrast, the data on mussels from the temperate seas are extensive. They generally indicate a decline in abundance in recent decades (Jones et al. 2010; Sorte et al. 2017; Seuront et al. 2019; Baden et al. 2021), which is consistent with the idea that warming should have a negative impact on the species in the “warmer” part of its range (Parmesan 2006).

**FIGURE 1:**
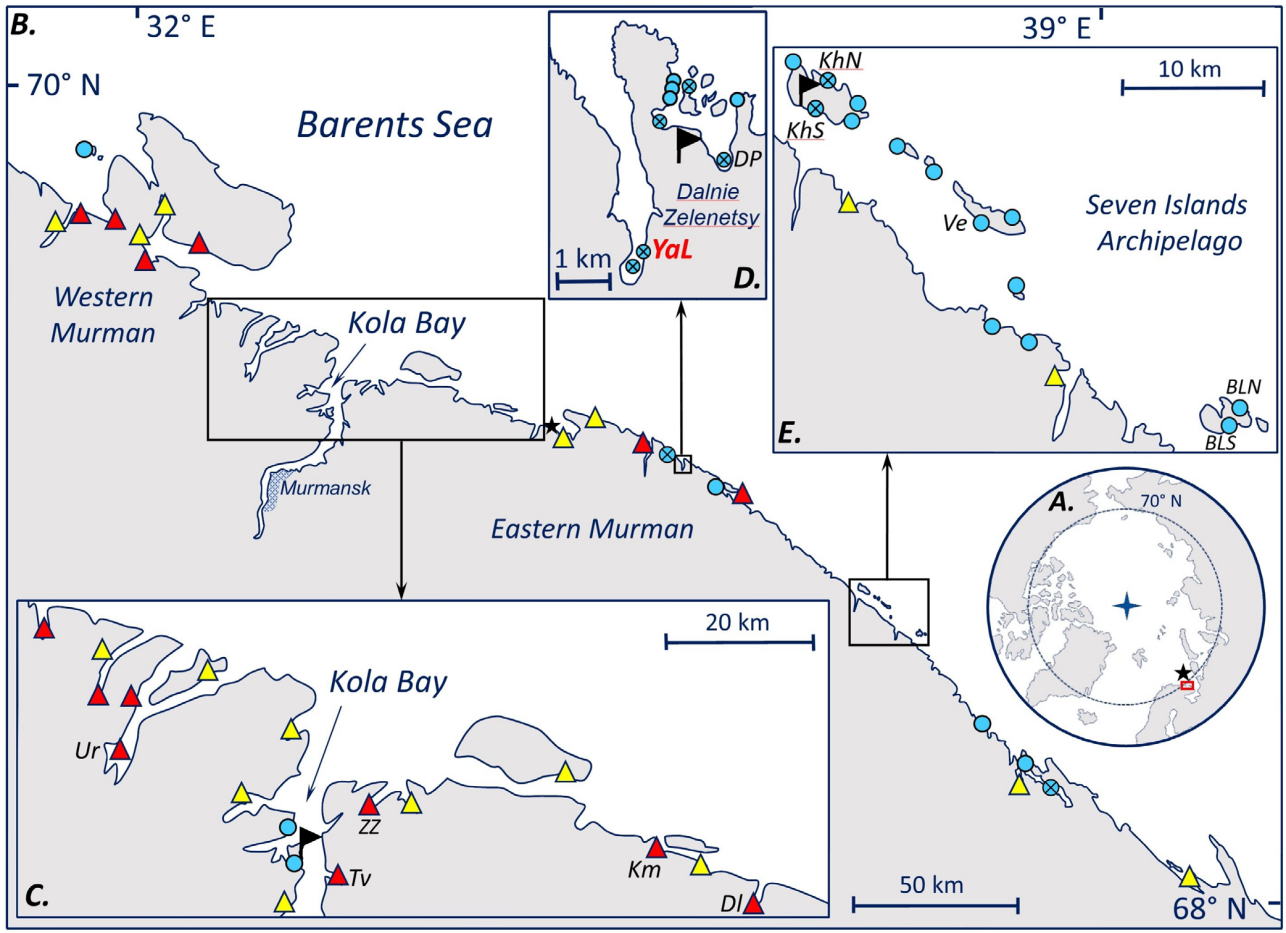
Sites of mussel surveys on the Murman Coast in 1933–2020. (А) Arctic. The location of the Murman Coast is indicated by the box. The star indicates stations 3–7 of the Kola Section, the temperature data from which were used in this study. (B) Murman Coast, areas of the most intensive mussel studies are indicated by boxes (C–E). The star shows the position of the weather station WMO no. 22028, the data from which were used in this study. (C) Outer Kola Bay and its vicinity. (D) Dalnie Zelentsy area. (E) Seven Islands Archipelago area. Flags in C–E denote research stations. Colored symbols in B–E mark the sites of mussel surveys. Triangles mark the sites of VNIRO surveys made in 1960–1961 (Romanova 1969, red and yellow filling) and in 1971 and 1981 (Antipova et al. 1984, yellow filling). All the sites surveyed in 1971 and 1981 were also investigated in the 1960s. Circles with crosses indicate our study sites. Monitoring sites where mussels were studied in at least three different decades are marked with their abbreviated names.

The Murman Coast, where our study was carried out, lies in the southwest of the Barents Sea, about 1000 km southeast of Spitsbergen (Figure 1). Even before the acceleration of the climate warming in recent decades, the thermal state of the Barents Sea has been unstable at the interannual and the decadal time scale, mainly due to variability in the inflow of warm Atlantic water (Levitus et al. 2009). These variations have been monitored at the Kola Section, with the oceanographic data from the 0 to 200 m water layer at stations 3–7 (Figure 1) being available since 1900 (Karsakov et al. 2022). These data are believed to be a reliable representation of the thermal dynamics of the entire Barents Sea (Fossheim et al. 2015; Dalpadado et al. 2020). Throughout the observation time, the coldest periods were 1900–1920s and 1960s–1980s, while the warmest periods were the very last decades (Boitsov et al. 2012; Karsakov et al. 2022).

The entire ecosystem of the Barents Sea has been changing alongside with the climate (Loeng and Drinkwater 2007; Matishov et al. 2012; Ingvaldsen et al. 2021; Pedersen et al. 2021). The general trend in the abundance of boreal species in 1950–2013 is U‐shaped, with low values during the coldest period in the 1960s–1980s (Pedersen et al. 2021). This trend is consistent with the data on fluctuations of the water temperature and correlated factors including primary production (Dalpadado et al. 2020). The available limited data for the first half of the 20th century also indicate that boreal species thrived during warm periods (Nesis 1960; Loeng and Drinkwater 2007 and references therein).

The Murman Coast is the northeasternmost edge of the Atlantic littoral communities and the only area of the Arctic Ocean which is usually free of ice throughout the year (Zenkevich 1963). For a long time the mussels in the Barents Sea were thought to be *M. edulis* but both *M. edulis* and *M. trossulus* were identified in genetic studies, the latter probably invading with maritime traffic in the 20th century (Väinölä and Strelkov 2011). With the exception of this invasion, the population dynamics of mussels at the Murman Coast can be considered as natural. Mussels are not fished or farmed there (Marchenko et al. 2023); in fact, direct anthropogenic impact is absent along most of the coast because human habitation is mainly concentrated in the inner part of the Kola Bay.

Despite a long history of quantitative studies at the Murman littoral (Zatzepin et al. 1948), including focused studies of mussels (Matveeva 1948; Antipova et al. 1984; Strelkov et al. 2001), data on the long‐term dynamics have never been summarized for any of the littoral species. We only know that the abundance of mussels declined dramatically along the entire coast in the 1960s (Antipova et al. 1984; Marchenko et al. 2022). This population collapse coincided with the start of the cold period (1960s–1980s) and was explained by the negative impact of the cooling (Antipova et al. 1984). This explanation seemed obvious, especially considering that the abundance of many other boreal species, including benthic ones, decreased in the Barents Sea at that time (Antipova 1975; see also above). However, in 2009 we re‐studied the mussel populations at the three “historical” sites and found that they had not recovered from the decline, even though the mean annual water temperatures in the Barents Sea had already exceeded the historical maximum by that time (Marchenko et al. 2023). This unexpected observation was one of the stimuli for the present study.

Our overall aim was to elucidate the patterns and drivers of the dynamics of subarctic mussels on the decadal scale using evidence from the Murman Coast. To achieve this aim, we tested two working hypotheses. Firstly, we summarized the available data on mussel abundance and assessed its association through time with water temperature at the Kola Section. We expected a positive relationship between abundance and temperature and a U‐shaped pattern of abundance in 1930s–2020s. Secondly, we determined the age structure of mussels in samples from the population monitored almost annually in 2001–2020, evaluated the success of different year‐of‐birth cohorts and correlated it with water temperature and other meteorological data from the nearest weather station. Using these data, we tested the hypothesis that mussels showed a more successful recruitment and/or survival in warm years than in cold ones.

## 2 Materials and Methods

### 2.1 Primary Data and the Workflow of the Analysis

#### 2.1.1 Analysis of Long‐Term Dynamics

The data in this study were derived from two sources: literature and our own material. As a measure of mussel abundance, we always employed mean biomass, kg/m^2^, calculated from samples collected on vertical transects (i.e., perpendicular to the shore) across the littoral at sites with known coordinates. Mussels from the same site are referred to in our study as a population. We used transect data because mussels are not uniformly distributed across the littoral and single or non‐random estimates may be biased. We used biomass rather than density because biomass is less sensitive to the accuracy of counting small mussels and because historical studies usually report biomass.

We searched for sources published before 2005 in the catalogs of the libraries of the Russian Academy of Sciences, the Kandalaksha State Nature Reserve and the St. Petersburg State University. Recent sources were looked for in Google Scholar with the keywords “*Mytilus*” and “Kola Bay” or “Barents Sea”. Our own data, mostly collected at the historical study sites, included 43 estimates from nine sites obtained in summer seasons 2000–2020 (Figure 1, Table S1). Samples, 3–5 per transect, were taken using a core of 0.01–0.1 m^2^ (the core area depended on the abundance of mussels) and washed through a 1.0 mm sieve. Biomass was either determined by weighing samples to the nearest 1 g or predicted from lengths of individual mollusks using the formula developed for the mussels at the Murman Coast in summer (Kostylev 1989). The latter method was used for the samples that had not been weighed immediately after collection (they were preserved as fixed material or dry shells). A comparison of biomass estimated directly and predicted from shell length in the samples for which both estimates were available showed that there were no differences in the results obtained with the use of these two methods. The details of sample collection and processing are provided in Table S1.

The general structure of the data was as follows. Several biological research stations operated along the coast at different time periods (Figure 1). Mussel populations near them, namely, the outer Kola Bay and its vicinity, Dalnie Zelentsy area and the Seven Islands Archipelago area (Figure 1), have been studied best and, at some sites, repeatedly (Strelkov et al. 2001; Marchenko et al. 2023). The populations studied in at least three different decades are referred to as monitoring ones. The Research Institute of Fisheries and Oceanography (VNIRO) surveyed mussel abundance along the coast in 1960–1961 (Romanova 1969), in 1971 and 1981 (Antipova et al. 1984), and in 2002–2005 (Milyutin and Sokolov 2006). During these surveys many sites were sampled in the same season or several consecutive ones (Figure 1).

In the data used in our study, different time periods were characterized by widely different numbers of biomass estimates, sometimes obtained at weakly overlapping sets of sites (see Section 3). For this reason, we chose to analyze them in a piecemeal fashion rather than as a whole. The following analyses were performed.

Firstly, we compared the patterns of biomass distribution along the coast in VNIRO surveys of different years (Model 1; Details of the fitting of all models are provided in the Section 2.2). In this way, we could take into account the heterogeneity of environmental conditions along the coast, in particular, the temperature gradient: the mean annual water surface temperature in the east is about 1.5°C lower than in the west (Terziev et al. 1990). Since the coast extends longitudinally (31.1°E–39.6°E), the differences in longitude between the survey sites were used as a proxy of the distance between them.

Secondly, we used the data from the best studied areas to reconstruct mussel dynamics there (Model 2). Finally, we compared the data from monitoring populations with water temperature. Data for two time intervals were analyzed: 1930s–1970s and 1980s–2010s (Model 3). The first interval covered the period from the beginning of observations to the collapse of the populations (Antipova et al. 1984; see also Section 3), while the second one covered all the subsequent years. We used the mean annual water temperatures in the 0–200 m layer at stations 3–7 of the Kola Section (Bochkov 2005; Karsakov et al. 2022; http://pinro.vniro.ru/ru/) averaged over the five years preceding the year of the mussel survey. It should be noted that there are no other data on water temperature for the entire period of interest (Karsakov et al. 2022). However, the analysis of temperature data at different depths at these stations for 1951–2017 has shown that fluctuations in annual water temperature in the 0–200 layer can be reliably extrapolated to the water surface (Boitsov and Guzenko 2021). The 5‐year period was chosen because local littoral populations are dominated by mussels under 6 years of age (Marchenko et al. 2023). This was further confirmed by the analysis of the data on *YaL* population (see below), where 1–5 year old mussels on average made up to 90% of the total biomass in the samples. For comparison, the contribution of 1–4 year old mussels was 80%.

#### 2.1.2 Demographic Analysis

Demographic analysis was performed based on the data from *YaL* population (Figure 1C), which was sampled in 2001–2007, 2009–2013, 2016–2018, and 2020. The choice of this population was associated with its accessibility (it is located near the biological station) and the abundance of historical data for it, dating back to the 1930s (Matveeva 1948), the 1970s and the 1980s (Strelkov et al. 2001). The age of all mussels in the samples or, if mussels in the samples were very numerous, in random subsamples from the samples (see Table S1), was assessed by counting marks of winter growth delay on shells as in Sukhotin et al. (2007). Numbers of mussels of individual age‐of‐birth cohorts (hereafter, generations) per m^2^ in different years were estimated.

Only generations born after 1998 and recorded in samples from at least four different years at an age older than one year were included in the analysis. Estimates of abundance in four different years were accepted as minimally sufficient to reconstruct the survivorship curve for a cohort. One‐year‐old mussels were ignored because their length could be less than 1 mm, which was the size of the mesh of the sieve used in our study.

We assumed that, since mussels are organisms with survivorship curve Type III (Deevey Jr. 1947), their numbers per m^2^ in individual generations should decline exponentially with time. This was indeed observed (see Section 3). Variations of logarithmic values of numbers of individual generations between years were approximated by the linear model. The slope of the regression (*z*) was considered as a proxy of the generation death rate per year while the intercept term (*N*_0_) was considered as a proxy of its recruitment success. For further analyses, each generation was characterized by the set of demographic parameters: *N*_0_, *z* and logarithmic values of mussel abundance at ages 2–10 (*N*_2_–*N*_10_). A few missing empirical values, that is, abundances of age cohorts absent in the samples due to limited sampling, were predicted from the model fitted. Mussels older than 10 years were extremely rare in our collections and therefore were ignored.

We set out to determine which environmental parameters available from the nearest weather station (Teriberka, WMO no. 22028, 40 km west from *YaL*, Figure 1) at http://portal.esimo.ru/ best explained the variation of demographic parameters between generations. The environmental parameters included air and sea surface temperatures, wave height, wind speed above water surface averaged over each of the four seasons of the year of generation birth, and air and sea surface temperatures averaged over each of the four seasons over the period from the year of generation birth to the year when it was recorded in our collections for the last time (data in Table S2). Wave height and wind speed were included because the intensity of water movement can affect the feeding and hence reproductive success of filter feeders. The seasons for the Barents Sea were defined following Terziev et al. (1990): winter in November–April (i.e., including the last two months of the previous year), spring in May–June, summer in July–August and autumn in September–October.

The workflow of the statistical analysis was as follows. Firstly, we identified the subset of environmental parameters that best explained the variation of demographic parameters between generations using the BIO‐ENV procedure (Clarke and Ainsworth 1993). It is based on comparing the biotic (in our case, demographic) similarity matrix with the equivalent matrices for all combinations of environmental parameters and selecting the subset of environmental parameters providing the best match between the two configurations. Secondly, we visualized and statistically evaluated the associations between demographic parameters using the same similarity matrix as in the first analysis and environmental parameters selected by BIO‐ENV with the help of redundancy analysis (RDA, van den Wollenberg 1977).

### 2.2 Statistical Modeling

The statistical analyses were carried out using the R statistical programming language (R Core Team 2025). Functions from the package “mgcv” (Wood 2011) were used for analyses of the long‐term mussel dynamics, and functions from the package “vegan” (Oksanen et al. 2013) were used for the demographic analysis (BIO‐ENV, RDA).

#### 2.2.1 Analysis of Long‐Term Dynamics

Generalized additive (mixed) models [GA(M)M, normal distribution] with raw biomass after Box‐Cox transformation (*λ* = 0.22) as a dependent variable were constructed. The Box‐Cox transformation was needed to symmetrise biomass distributions (Asar et al. 2017). Its parameters were found using the package “aid” (Asar et al. 2017). The validity of each GA(M)M model was checked by visual analysis of residual plots. Three regression models were fitted.

**Model 1**: Biomass as a function of longitude in VNIRO surveys of different years. GAM with *Longitude* (continuous predictor) and *Survey* (discrete predictor with four levels) was fitted. The smoothers for each *Survey* were fitted separately.

**Model 2**: Biomass as a function of the year of survey in the best studied areas. GAMM with *Year* (continuous predictor) and *Area* (discrete predictor with three levels) was fitted. *Site* was included as a random factor. The smoothers for each *Area* were fitted separately.

**Model 3**: Biomass in monitoring populations as a function of water temperature in two time periods, 1930s–1970s and 1980s–2010s. GAMM with *Temperature* (continuous predictor) and *Period* (discrete predictor with two levels) was fitted. *Site* was included as a random factor. The smoothers for each *Period* were fitted separately.

#### 2.2.2 Demographic Analysis

In the BIO‐ENV procedure the demographic matrix with rows corresponding to years of generation birth and demographic parameters as columns was transformed into a matrix of Euclidean distances between years. This matrix was compared using Mantel correlation with 16,777,215 matrices of Euclidean distances between years, derived from all possible subsets of 24 environmental parameters.

The statistical significance of the RDA model, individual canonical axes and environmental constraints was assessed by permutation methods (Borcard et al. 2011), with 9999 permutations being set up.

## 3 Results

### 3.1 Long‐Term Dynamics

In total, we obtained 173 estimates of mean biomass at 62 sites from 1933 to 2020 including 130 estimates at 60 sites derived from literature (Table S3). The most abundant data are available for the 1960s, while data for the 1940s and the 1990s are completely missing. By the number of data sources, the 1930s, the 1960s, the 1970s, the 1980s and the 2000s are represented by four sources each, the 2010s by two sources, and the 1950s by only one source. It is also worth mentioning that in the 2010s, the vast majority of collections were made by the authors of this study, while in the 1960s and 1980s the majority of collections were made during VNIRO surveys (Romanova 1969; Antipova et al. 1984) (Table 1; Table S3). Twelve populations from the three best studied areas (the outer Kola Bay and its vicinity, Dalnie Zelentsy area and the Seven Islands Archipelago area) were studied in at least three different decades and are referred to as monitoring populations (Figure 1; Table S3).

**TABLE 1:** Structure of the data on the abundance of Murman mussels in 1933–2020.

Decade	KB	DZ	SI	Total	References
1930–1939	2/2 *1.2 ± 0.3*	8/8 *7.8 ± 3.37*	9/9 *3.2 ± 0.66*	19/19 *4.9 ± 1.52*	Zatzepin et al. (1948), Gurieva (1948), Matveeva (1948), and Raskina (1963)
1950–1959	—	—	7/7 *0.06 ± 0.02*	8/8 *0.08 ± 0.03*	Kussakin (1963)
1960–1969	20/16 *8.9 ± 1.86*	—	15/14 *0.3 ± 0.16*	54/45 *5.3 ± 0.88*	Karpovich (1967), Karpovich (1968), Karpovich (1969), and Romanova (1969)
1970–1979	6/6 *1.0 ± 0.37*	4/2 *0.7 ± 0.40*	15/3 *0.2 ± 0.09*	27/13 *0.5 ± 0.13*	Streltsov et al. (1978), Antipova et al. (1984), Karpovich and Shklyarevich (1997), and Strelkov et al. (2001)
1980–1989	4/3 *0.8 ± 0.55*	1/1 *1.7*	—	10/9 *0.6 ± 0.27*	Antipova et al. (1984), Filippov (1986), Kostylev (1989), and Strelkov et al. (2001)
2000–2009	4/4 *1.5 ± 0.45*	24(22)/5(5) *3.0 ± 0.73*	3 (2)/3 (2) *1.4 ± 0.49*	37(26)/18(9) *2.4 ± 0.49*	Strelkov et al. (2001), Milyutin and Sokolov (2006), and Marchenko et al. (2023)
2010–2020	1/1 *1.6*	17(17)/3(3) *2.7 ± 0.59*	—	18(17)/4(3) *2.6 ± 0.57*	Marchenko et al. (2023)
Total				173 (43)/62 *3.1 ± 0.37*	

*Note:* For each time period (decade), the number of observations at different sites in different years and, after the slash, the number of study sites at the entire Murman Coast and at each of the three best studied areas are given in the top rows. Study sites are shown in Figure 1. Three best studied areas are the outer Kola Bay and its vicinity (KB), Dalnie Zelentsy (DZ) and the Seven Islands Archipelago (SI). Since some sites were studied repeatedly within the same decade, the number of observations usually exceeds the number of sites. Our data (in parentheses) are given separately from the published data; references to published data are provided. Mean biomasses ± Standard Errors of means for corresponding sets of observations are given in italic in the bottom rows.

**FIGURE 2:**
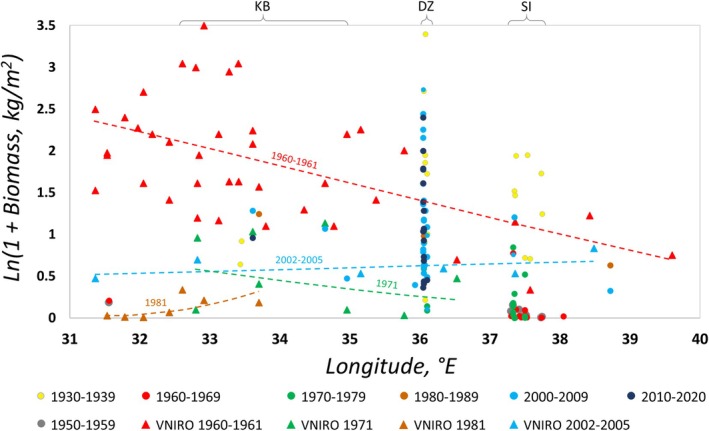
Distribution of mussel biomass along the Murman Coast in different decades. The colors of the symbols indicate estimates obtained in different time periods, triangles show data from VNIRO surveys and dots show data from other studies (see legend). Lines are GAM predictions fitted to VNIRO data from different surveys (Model 1). The positions of the best studied areas, labeled as in Table 1, are indicated in the top of the figure. Here and below the model predictions were log transformed after backward Box‐Cox transformation for better visualization.

A general idea of the main patterns of spatial and temporal dynamics of mussels at the Murman Coast can be derived from Figure 2, where biomasses obtained in different decades and during different VNIRO surveys are shown as a function of longitude. Two trends are noteworthy. Firstly, biomass tended to decrease with longitude in the data‐rich 1960s. Secondly, the overall decrease in biomass after the 1960s can be seen. Both trends are confirmed by statistical analysis of VNIRO data. The fitted GAM confirmed a negative dependence of biomass on longitude in the 1960–1961 survey, with a dozen kilograms per m^2^ in the west of the coast to a few kilograms in the east. It also confirmed that the biomasses in the 1960–1961 survey were higher than in all subsequent ones. In 1971, 1981 and 2002–2005 surveys biomasses were comparably low, on average less than 1 kg/m^2^, and were not associated with longitude (Model 1, Table S4; Figure 2).

**FIGURE 3:**
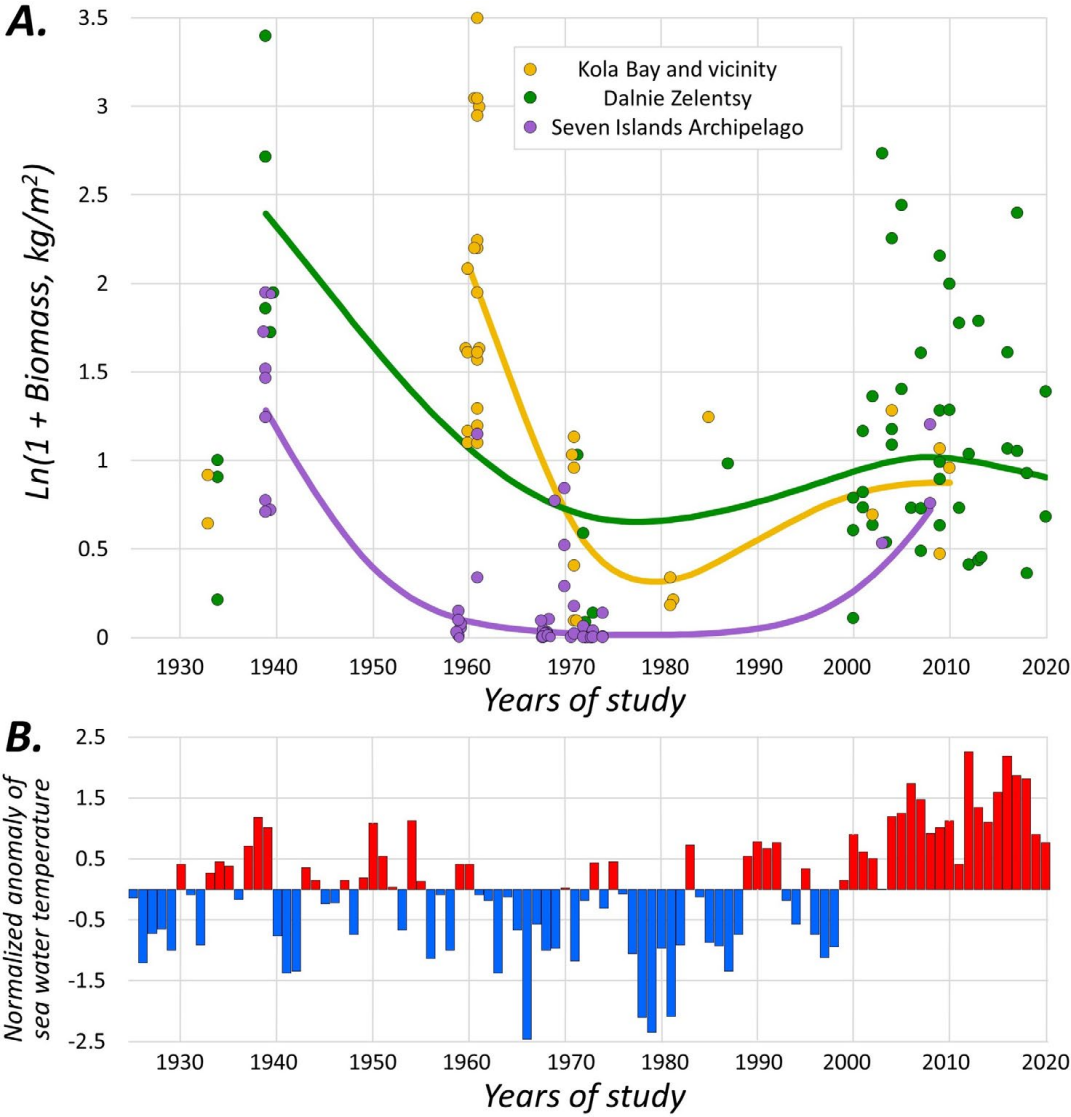
Long‐term changes of the biomass of littoral mussels at the three best studied areas of the Murman Coast and of annual water temperature in the Barents Sea. (A) Biomass, log kg/m^2^. Dots—empirical estimates, lines—GAMM predictions for the entire period from 1939 to 2020 (Model 2). Data from different areas are shown by different colors (see legend). (B) Normalized anomalies of annual water temperature relative to the average of 4.15°C in 1925–2020 at 0–200 m depth at stations 3–7 of the Kola Section according to Bochkov (2005) and Karsakov et al. (2022).

The pattern of temporal dynamics of biomass in populations from the three best studied areas throughout the entire observation period was similar and could be characterized as L‐shaped rather than U‐shaped. The maximum values were observed closer to the beginning of observations in the 1930s–1960s, while the minimum values were observed in the 1970s–1990s; in comparison, the values in the 21st century were medium‐low (Figure 3A). It should be noted that the biomass was also low in the early 1930s and the late 1950s (Figure 3A), but the data for these periods are scarce and appear to be insufficient for conclusions.

The fitted GAMM confirmed that the curvilinear pattern of dynamics was similar in all the areas; it also confirmed that the biomass in the Seven Islands Archipelago area was generally lower than in the other areas (Model 2; Table S4).

Annual water temperature variation throughout the observation period was an inverted image of that of the biomass: the first decades of the 21st century were extremely warm, the 1970–1990 were the coldest, and the 1930–1960 were relatively warm (Figure 3B). This means that the biomass was very high during the relatively warm 1930s–1960s, extremely low during the cooling of the 1960s–1990s, and moderately low during the recent warming, which is the most significant in recorded history.

**FIGURE 4:**
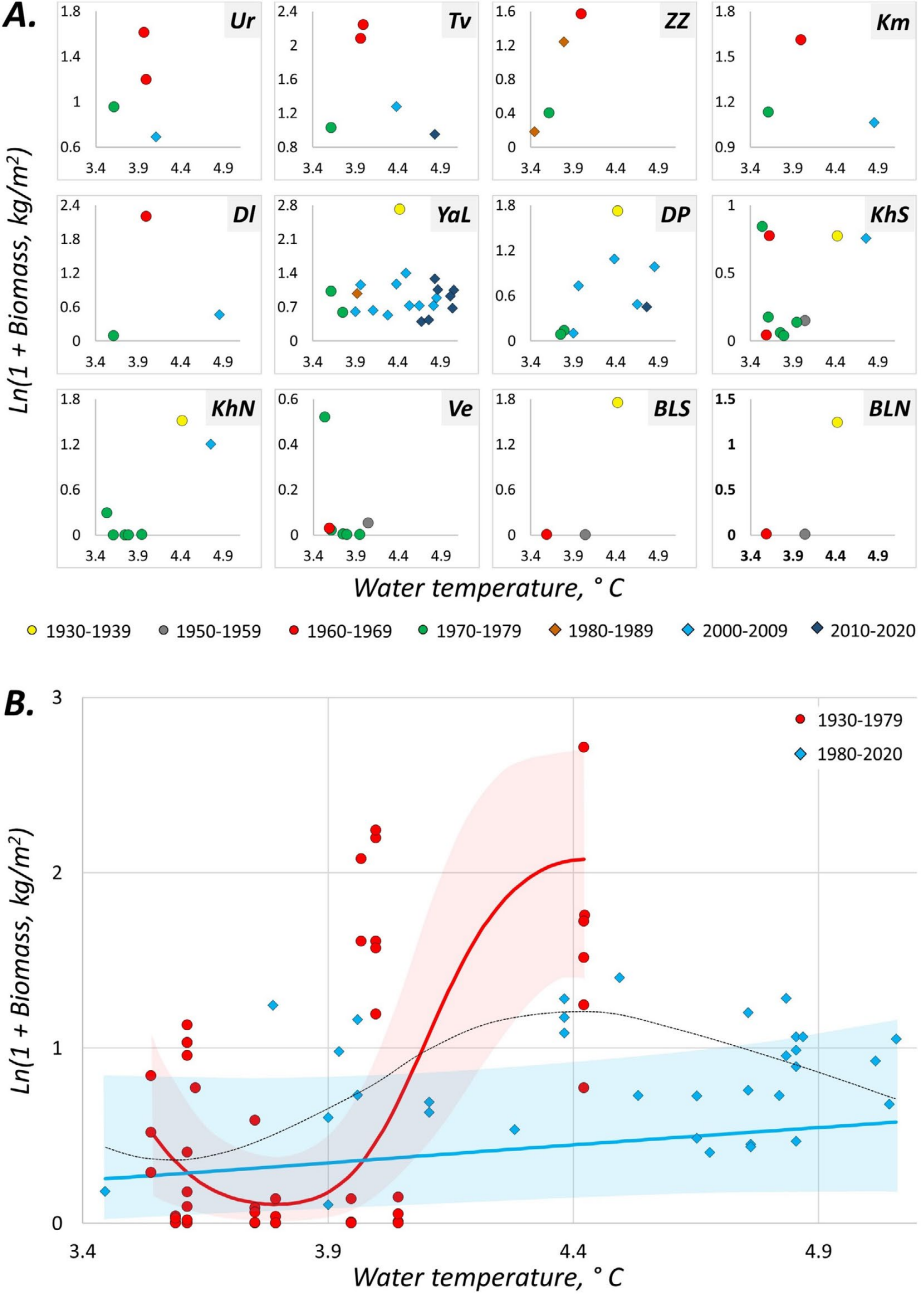
Relationship between mussel biomass in the monitoring populations in different years and water temperature. Temperature at 0–200 m depth at stations 3–7 of the Kola Section averaged over five years preceding the year of the mussel survey is used. (A) Primary data. Abbreviated site names are as in Figure 1 and Table S3. Estimates from different decades are shown by different colors (see legend). (B) Predictions of regression models. Dots are all biomass estimates in monitoring sites. Solid lines are GAMM predictions fitted to data from different periods (see legend) (Model 3). Black dashed line is LOESS approximation of all the data.

The relationship between the biomass of mussels in the monitoring populations and water temperature averaged over the five years preceding the year of the survey can be derived from Figure 4. Visual inspection of the primary data (Figure 4A) shows that (a) in all the cases the maximum biomass was observed in the 20th century, mostly in the 1930s or the 1960s, (b) in all the cases except one (population *Ve*) there was a trend toward a positive relationship between biomass and temperature in the 20th century, due to the differences between biomass estimates made during periods with contrasting temperature before and after the early 1960s, and (c) this relationship became blurred after the addition of the data from the 21st century. In particular, no trend was observed in the best studied *YaL* population in 1970–2020.

These trends were confirmed by GAMM. There was a strong positive relationship between the biomass and water temperature in the 1930s–1970s; after that, the relationship, though positive, was weak and not statistically significant (Figure 4B; Model 3, Table S4). Throughout the observation period, the relationship was dome‐shaped, with the maximum expected biomass values at temperature around 4.4°C, which is close to the long‐term average of 4.15°C (Figure 4B).

### 3.2 Demographic Analysis

**FIGURE 5:**
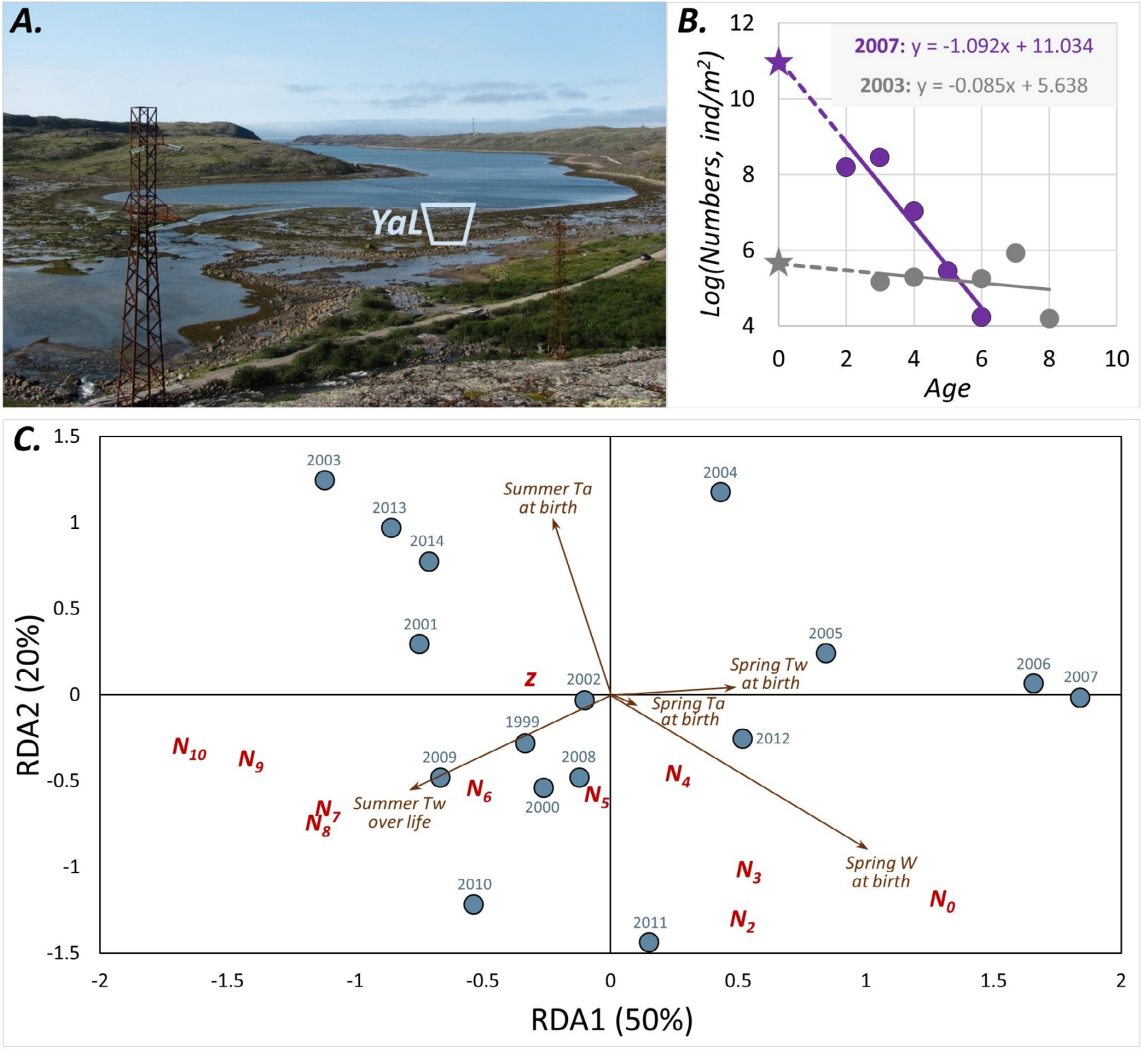
Influence of environmental factors on the abundances of mussels of different generations in *YaL* population in 1999–2020. (A) Panorama of the top of Yarnyshnaya Inlet at low tide. The photo was taken from the southern shore in 2013. The study site (*YaL*) is marked with a trapezium. Width of the mussel‐inhabited littoral zone is 150 m. (B) Two contrasting survivorship curves for generations of 2003 and 2007. Dots are numbers, lines are regressions. Stars label *N*_0_. Equations of linear models (*y* = *z* × *x* + *N*_0_) are given. (C) Redundancy analysis (RDA) ordination plot showing the relationship between generations characterized by demographic parameters (*N*_0_, *z*, numbers in different years throughout the lifetime) and environmental constraints selected by BIO‐ENV as best explaining the variation between generations (see the text). Points represent generations (years of birth are indicated). Text markers represent demographic parameters. Arrows indicate environmental constraints. *Ta* and *Tw* stand for air and water temperature, respectively, *W* stands for wind speed.

Sixteen generations born between 1999 and 2014 met data completeness requirements and were included in the analysis. Logarithmic values of numbers of individual generations generally decreased with age in a linear manner (Figure S1; Table S5; Figure 5B), and so it was possible to consider the slope of the linear regression (*z*) as a proxy of the generation death rate per year and the intercept term (*N*_0_) as a proxy of its abundance at start, the that is, recruitment success. There was (1) a strong positive correlation between *N*_0_ and numbers of younger mussels (*N*_2_–*N*_4_) within generations as well as (2) a strong negative correlation of *N*_0_ and *z* between generations (Figure S2, see also below), indicating that mortality was density‐dependent.

The results of BIO‐ENV are illustrated in terms of RDA (Figure 5C); full results are provided in Table S6. The first two canonical axes (RDA1, RDA2) were statistically significant (*p* < 0.05), and so was the model as a whole (Table S7). Out of the five environmental parameters identified by BIO‐ENV as best explaining the variation between generations, the wind speed and surface water temperature in spring of the year of birth and summer surface water temperature averaged over the lifespan significantly affected the ordination while the effects of air temperature in summer and air temperature in spring of the year of birth were marginally significant (0.05 < *p* < 0.1, Table S7).

Out of the demographic parameters associated with RDA1, which explained 50% of inertia, *N*_0_ and *N*_2_–*N*_4_ showed a positive association, while *z* and *N*_5_–*N*_10_ showed a negative association (Figure 5C). Out of the environmental parameters correlated with this axis, wind speed, water and air temperatures in spring of the year of birth were positively correlated while summer water temperature during the lifespan was negatively correlated. That is, survivorship was positively associated with water temperature in summer (note that *z* has a negative sign) while the recruitment was positively associated with temperature and wind speed in spring (see Figure S3 for visualization of associations between *N*_0_, *z* and environmental parameters).

All demographic parameters except *z* were negatively associated with RDA2, indicating that this axis reflected the overall abundance throughout the lifespan. Only air temperature in the summer of the year of birth was positively correlated with RDA2. Since this parameter weakly affected the ordination and since RDA2 explained only 20% of inertia, this axis can be considered as uninformative.

## 4 Discussion

In this study we tested the hypothesis that climate warming should have a favorable effect on littoral mussels in the subarctic Barents Sea. We gathered and analyzed two datasets: one on biomass dynamics along the entire Murman Coast from the late 1930s to 2020 and the other on the age structure of *YaL* population in the middle of the coast in 2001–2020. In terms of time span and detail, these data series are comparable with exemplary monitoring population studies of mussels and other littoral animals in temperate seas (Sagarin et al. 1999; Hawkins et al. 2008; Menge et al. 2009; Beukema et al. 2015; Firth et al. 2015; Sorte et al. 2017; Petraitis and Dudgeon 2020; Beukema and Dekker 2020; Little et al. 2024). As for the subarctic, these data series are unprecedentedly extensive (see Dornelas et al. 2025 for review).

The analysis of the *YaL* data suggests that *YaL* was a stable population in 2001–2020 where abundance varied in a limited range (Figure 4A). An increased abundance of certain generations at the start (predicted recruitment success, *N*_0_, which was directly proportional to the observed abundance of generations at the youngest ages) was mostly offset by their increased elimination with age (death rate, *z*). These observations indicate a density‐dependent mortality.

Comparison of *N*_0_ and *z* values for different generations with environmental conditions in the years of their birth and during their lifetime, respectively, revealed positive correlations of *N*_0_ with water temperature and wind speed in spring as well as positive correlations of *z* with water temperature in summer. These results suggest a positive effect of temperature on recruitment and survival of mussels. Besides, strong wind currents ensure good food supply for filter feeders. In spring, which is the time of gametogenesis (Maximovich 1985), the combination of elevated temperature and strong winds may promote successful maturation of gametes.

The *YaL* data indirectly suggest a positive relationship between temperature and mussel recruitment in the subarctic. In the “warmer” part of boreal mussels' ranges, an opposite correlation is observed. Variability in mussel recruitment over decades has been monitored in Oregon (Menge et al. 2009), the Gulf of Maine (Petraitis and Dudgeon 2020) and the Wadden Sea (Beukema et al. 2015). All these studies suggest a negative relationship between temperature and recruitment. Temperature affects recruitment of mussels both directly and indirectly, through more temperature‐sensitive forage phytoplankton and predators (Menge et al. 2009; Beukema et al. 2015; Petraitis and Dudgeon 2020). However, since the average annual water temperatures in the waters off the Murman Coast are 3–6 degrees lower than in Oregon, the Wadden Sea and the Gulf of Maine (www.seatemperature.info), the mechanisms uncovered in these areas are probably not directly applicable to Murman mussels. Besides, predation is unlikely to play any significant role at the Barents Sea littoral since mussel predators are scarce there (Marchenko et al. 2023).

Surprisingly, the abundance of mussels in the *YaL* population was much higher 60–80 years before our observations, when the temperatures were lower (Matveeva 1948; Figure 4A). The same situation was observed elsewhere along the Murman Coast. No apparent explanation of this finding can be derived from the *YaL* dataset.

The analysis of the dataset on long‐term biomass dynamics provided the following findings. Firstly, and most importantly, the mussel abundance along the Murman Coast was generally high until the early 1960s and generally low onwards. The collapse of populations in the 1960s has been noted in all mussel studies from that time, including those whose data are included in our analysis (Streltsov et al. 1978; Antipova et al. 1984; Karpovich and Shklyarevich 1997) as well as those that were not included because did not contain biomass data (Agarova et al. 1976). Thus, the collapse can be considered as an established fact.

The second result is that there was a trend toward recovery in the period after the collapse. In the 1980s the mean biomass was about 10% of that in the early 1960s, while in 2010s it was already about 50%. Unfortunately, we cannot be sure when the recovery started because of the paucity of the data for the 1980s and 1990s. However, our own unpublished observations indicate that there were an order of magnitude fewer mussels in the Dalnie Zelentsy area (see Figure 1) in the early 1990s than in the 2000–2020s. Thirdly, we revealed contrasting patterns in the spatial distribution of the mussel biomass in the large‐scale VNIRO surveys of 1960–1961 (i.e., shortly before the collapse) and in later times. In the 1960–1961 survey the biomass decreased from west to east, while in subsequent decades no spatial patterns were observed.

The fourth finding is the lack of a temporal correlation between the mussel biomass and water temperature (averaged over the five years preceding the year of the survey) in the period after the collapse. This is all the more surprising given that this period included both very cold decades in the 20th century and extremely warm last decades. Even during our own observations (2001–2020), the average annual temperature rose by more than one degree Celsius, but it had little effect on the abundance of mussels (Figure 4A). The only indications of a positive relationship between mussel abundance and temperature in the data are the decrease of the mussel biomass with longitude in the 1960–1961 VNIRO survey, which parallels the decrease in the water temperature (Terziev et al. 1990), and the fact that the collapse coincided with the cooling of the Barents Sea.

It should be noted that the 1960s–1980s were not the only cold period in the 20th century. There was another cooling in the 1900s–1920s. No quantitative mussel surveys were conducted at that time. However, it would not be unreasonable to conjecture that this cold period had no long‐term effect on the mussel abundance because it was quite high in the late 1930s (Figure 3).

To the best of our knowledge, no trends similar to those uncovered in our study have been previously recorded in other long‐term surveys of the abundance of mussels or any other littoral animals. In most of them interannual fluctuations and/or long‐term trends were at least partly consistent with climatic trends (Sagarin et al. 1999; Hawkins et al. 2008; Firth et al. 2015; Westerbom et al. 2019; Beukema and Dekker 2020; Baden et al. 2021).

In summary, the 1960s were the tipping point in the dynamics of the mussel populations at the Murman Coast over the period from the 1930s to the 2020s. A dramatic collapse occurred at that time, and it is unclear whether the populations have stabilized or are still recovering. Below we discuss the possible causes of the collapse of the mussel populations at the Murman Coast in the 1960s and of their poor recovery afterwards.

### 4.1 Collapse

We do not know what caused the collapse of the mussels populations at the Murman Coast in the 1960s and whether the decline was abrupt or gradual. It has been suggested that the low temperatures negatively affected mussel reproduction and/or larval survival (Antipova et al. 1984; Strelkov et al. 2001). This hypothesis still seems reasonable. In the subarctic, mussels spawn once a year synchronically in local populations and at the same temperatures, and larval development coincides with the phytoplankton bloom (Maximovich 1985; Maximovich and Shilin 2012). In the Murman waters, mussels spawn in July at temperatures slightly below 10°C (Strelkov et al. 2001), while the phytoplankton bloom starts in spring and ends in early autumn (Dalpadado et al. 2020). Survival of developing larvae depends on the abundance of phytoplankton (Menge et al. 2009) and on the temperature: the lower the temperature, the longer the development and the higher the mortality (Bayne 1965).

This means that poor reproduction or recruitment of mussels in the cold years may be explained by several mechanisms. Firstly, the water may be not warm enough for spawning. Secondly, if the water warms later than usual in summer, larval development may be desynchronised with the phytoplankton bloom. Thirdly, larvae developing at lower temperatures may have poorer survival. Lastly, lower temperatures in spring can negatively affect gametogenesis, as our observations at *YaL* suggest.

The collapse of the mussel population could also be due to a mass mortality event caused, for example, by a new disease, as recently occurred in France (Benabdelmouna and Ledu 2016), or by a cold snap, as recently occurred in Canada (Cameron and Scrosati 2023). It is noteworthy that in the winter of 1962–1963, which was extremely cold everywhere in Europe, mass mortality of many littoral species was noted in the British Isles (Crisp 1964; Helmuth et al. 2006). This cold snap may have affected mussels in the Barents Sea, too. Other proposed causes of the decline of blue mussel populations in temperate seas—overfishing (Baden et al. 2021; Heard et al. 2025) and heat waves (Seuront et al. 2019)—are probably not valid for the Murman Coast. Mussels have never been fished there, while deadly heat waves appear improbable in the Barents Sea subarctic, especially during the cold decades of 1960s–1970s.

### 4.2 Poor Recovery

If low temperature was indeed the root cause of the decline of mussel populations in the 1960s, the unfavorable period lasted for about 30 years (Figure 3B). However, the cold period ended 30 years ago, and yet the populations have not fully recovered. To explain the poor recovery of mussel populations after the collapse in the 1960s and, in particular, their seeming insensitivity to the subsequent warming, we suggest two hypotheses that are not mutually exclusive.

The first hypothesis is that the mussel populations at the Murman Coast experienced the Allee effect. Once the population density dropped to a low level, the population growth was limited for generations. The Allee effect may be underlain by various mechanisms (reviewed in: Gascoigne and Lipcius 2004; Berec et al. 2007). Since mussels are sedentary broadcast spawners, two mechanisms seem particularly likely. The first is poor synchronization of spawning at low densities: individuals spaced far apart cannot effectively exchange chemical cues inducing spawning (Lundquist and Botsford 2011; Rato et al. 2025). The second is poor fertilization at low densities due to gamete dilution, especially considering that sperm are short‐lived (Levitan and Petersen 1995; Babcock and Keesing 1999). Theoretically, one more mechanism is known for bivalves: dense clumps of adults serve as shelters for juveniles, so that a shortage of adults translates into that of recruits (Fariñas‐Franco and Roberts 2018). However, subarctic *Mytilus* are not known to have such “nurseries” (Khaitov 2013), and this mechanism probably does not apply to them.

In our opinion, the Allee effect explains the poor recovery of Murman mussels after the collapse of the 1960s fairly well. A similar hypothesis has been invoked to explain poor recovery of overexploited populations of marine fish species (Keith and Hutchings 2012), many of which are broadcast spawners, too. The analysis suggests that marine fish populations depleted to less than 10% of maximum abundance, especially for lengthy periods of time, recover poorly even under optimal conditions (Hutchings 2015). This scenario agrees with our observations of the Murman mussels, especially if they were indeed depleted as a result of a prolonged cold spell.

The second hypothesis explaining the poor recovery of the mussel populations at the Murman Coast postulates that their taxonomic structure was different before and after the collapse (Marchenko et al. 2023). Before the collapse the mussels were represented exclusively by *M. edulis* , while afterwards the populations consisted of a mixture of *M. edulis* and *M. trossulus* . Competition and hybridization between these two cryptic species could limit the abundance. *Mytilus trossulus* in the Barents Sea is genetically similar to its source population in the West Atlantic (Väinölä and Strelkov 2011; Wenne et al. 2020; Simon et al. 2021). It is unlikely that it invaded the Murman Coast earlier than in the 20th century since there were no harbors there before the foundation of Murmansk in 1916. Väinölä and Strelkov (2011) suggested that *M. trossulus* was brought with Arctic convoys of WWII and that its naturalization was facilitated by the collapse of the native mussel populations in the 1960s. The limited available data indicate that *M. edulis* is generally more common at the Murman Coast, while *M. trossulus* and hybrids between the two species are usually found in samples together with *M. edulis* (Väinölä and Strelkov 2011; Khaitov et al. 2021; Marchenko et al. 2023). Reduced fitness of hybrids between *M. edulis* and *M. trossulus* (Kenchington et al. 2020) and competition between these two species (Khaitov et al. 2025) have been proved.

## 5 Conclusion

Climate warming has accelerated in the 21st century, affecting all components of the ecosystems. While boreal species generally experience population decline and range contraction in temperate latitudes, they thrive and expand their ranges in the Arctic. The latter trend, supported by the plethora of data for the Barents Sea and its vicinity, is indisputable (Fossheim et al. 2015; Ingvaldsen et al. 2021; Pedersen et al. 2021). Twenty years ago, blue mussels were the heralds of borealization in the Arctic, appearing on Spitsbergen for the first time after a thousand‐year absence (Berge et al. 2005).

In the light of this, the case of blue mussels at the Murman Coast is all the more intriguing. Their dynamics is inconsistent with the general tendency. We showed that mussels were abundant before the advent of the climate warming and scarce after that. Considering the ecological importance of mussels (Seed and Suchanek 1992), there are reasons to believe that this change in their dynamics is affecting many other species.

We hypothesize that the unusual pattern of long‐term dynamics of mussel populations at the Murman Coast may be due to some momentous events in the past, more precisely, in the cold decades of the 20th century. The population collapse in the 1960s, when mussel biomass dropped to approximately 10% of the pre‐collapse level, could be such an event. The Allee effect, possibly triggered by the collapse, may still be limiting the recovery. The invasion of *M. trossulus* in the mid‐century could be another event with far‐reaching consequences, bringing into play interspecific competition and hybridization that are likely to impair the population dynamics.

## Author Contributions

**Julia Marchenko:** conceptualization (equal), data curation (lead), formal analysis (lead), investigation (equal), methodology (equal), writing – original draft (equal), writing – review and editing (equal). **Vadim Khaitov:** formal analysis (equal), investigation (equal), methodology (equal), writing – original draft (equal), writing – review and editing (equal). **Larisa Basova:** investigation (equal), resources (lead). **Evgeny Genelt‐Yanovskiy:** conceptualization (equal), investigation (equal), writing – review and editing (equal). **Sergey Malavenda:** investigation (equal), writing – review and editing (equal). **Petr Strelkov:** conceptualization (equal), funding acquisition (lead), investigation (equal), methodology (equal), supervision (lead), writing – original draft (equal), writing – review and editing (equal).

## Funding

This study was supported by the Russian Science Foundation, Grant Number 26‐14‐00088.

## Conflicts of Interest

The authors declare no conflicts of interest.

## Supporting information


**Figure S1:** Survivorship curves for the 1999–2014 generations.
**Figure S2:** (A) Pearson's correlation coefficients between *N*_0_ and *N*_2_–*N*_10_ for all generations. (B) Association between *N*_0_ and *z* among generations.
**Figure S3:** The relationships between *N*_0_, *z* and selected environmental parameters.

**Table S1:** Information about mussel samples from our own surveys (2000–2020).

**Table S2:** Environmental data used in demographic analysis (BIO‐ENV and RDA).

**Table S3:** All data on mussel biomass used in a study.

**Table S4:** Parameters of the fitted regression models (GAM(M)).

**Table S5:** Parameters of individual mussel year‐of‐birth cohorts (generations) in the *YaL* population as used in BIO‐ENV and RDA.

**Table S6:** Results of BIO‐ENV analysis.

**Table S7:** Permutational assessment of redundancy analysis (RDA) results examining the association between demographic and environmental parameters of mussel generations in 1999–2014.

## Data Availability

All initial data and code to reproduce the results presented in main text of this manuscript are available through database of St. Petersburg State University (https://hdl.handle.net/11701/49666). The datasets and figures supporting this article have been uploaded as part of the Supporting Information.
