# Supplementary material for: The Mussels That Came in From the Cold: Long‐Term Effects of the Population Collapse in the 1960s May Explain Low Abundances of Boreal Mussels in the Subarctic Despite the Warming
Source: Ecol Evol. 2026 Jun 3;16(6):e73763. doi: 10.1002/ece3.73763 (PMC13240005; doi:10.1002/ece3.73763)
Supplement: Supplementary file 1 — Figure S1: Survivorship curves for the 1999–2014 generations. Figure S2: (A) Pearson's correlation coefficients between N 0 and N 2–N 10 for all generations. (B) Association between N 0 and z among generations. Figure S3: The relationships between N 0, z and selected environmental parameters. [file ECE3-16-e73763-s004.docx]

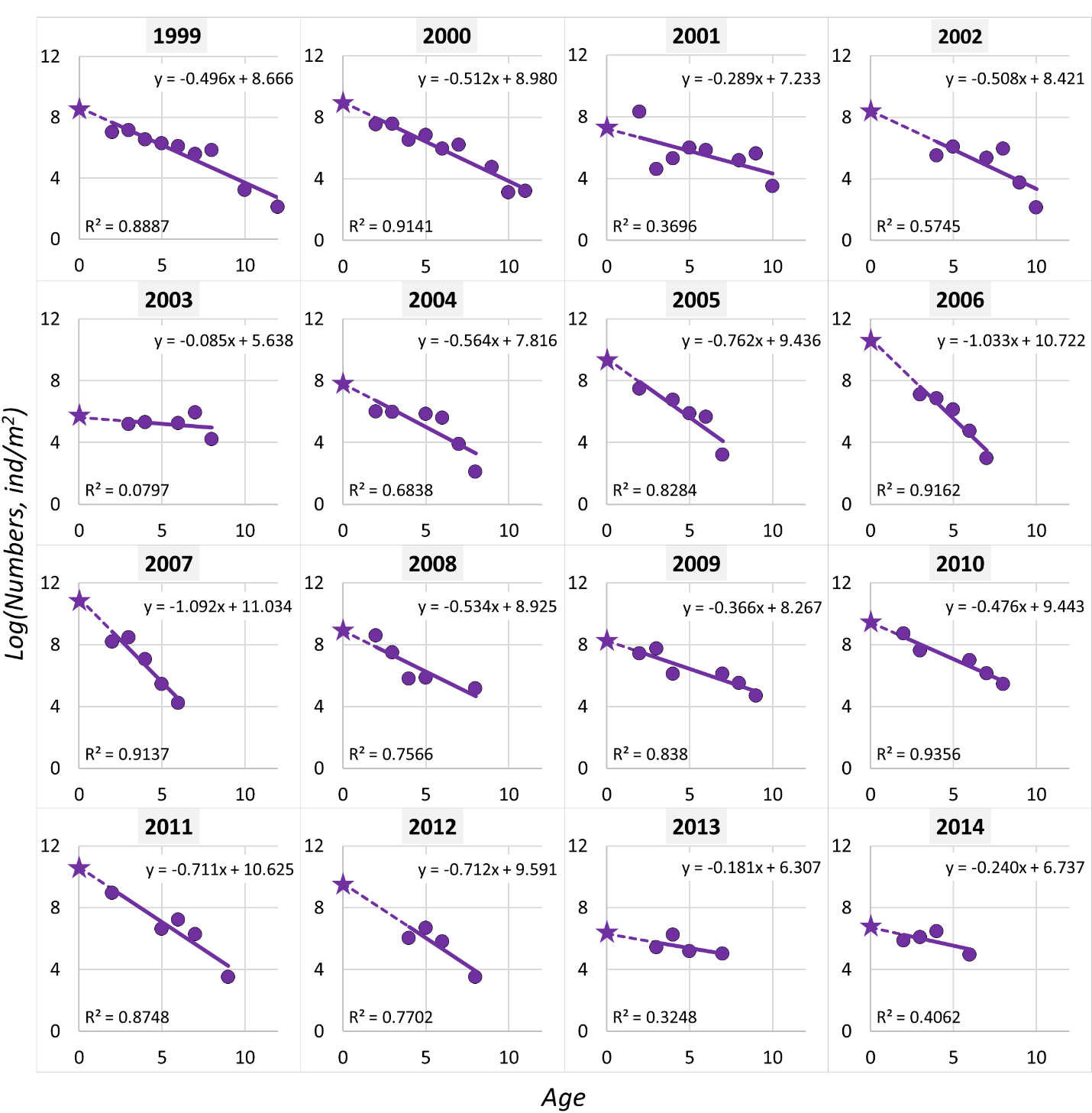


**Figure S1. Survivorship curves for the 1999-2014 generations**. Dots are observed numbers, lines are regressions. Asterisks label *N_0_*. Equations of linear models (y = *z**x + *N_0_*) and coefficients of determination (R^2^) are given.


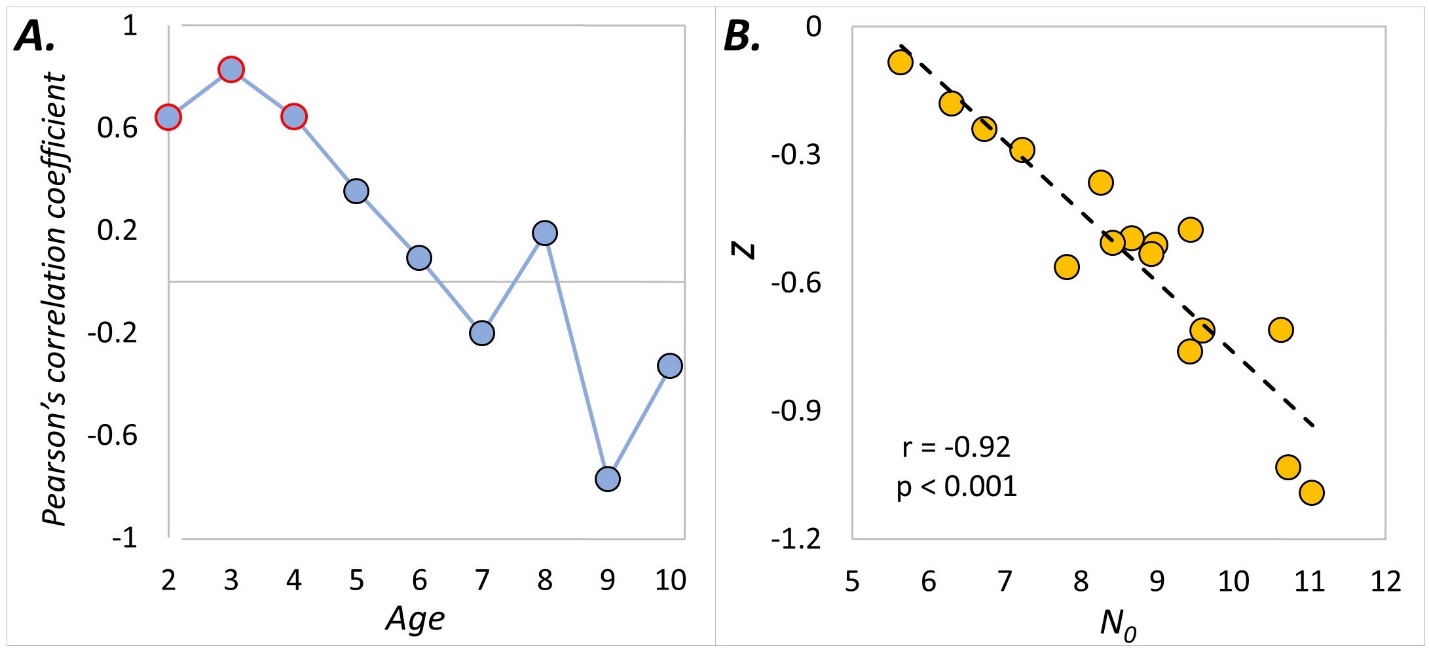


**Figure S2. (A)** Pearson’s correlation coefficients between *N_0_* and *N_2_-N_10_* for all generations. Significant correlations (p<0.05) are marked by symbols with red wrap. **(B)** Association between *N_0_* and *z* among generations. Pearson’s correlation coefficient (r) and p-value are given.

**
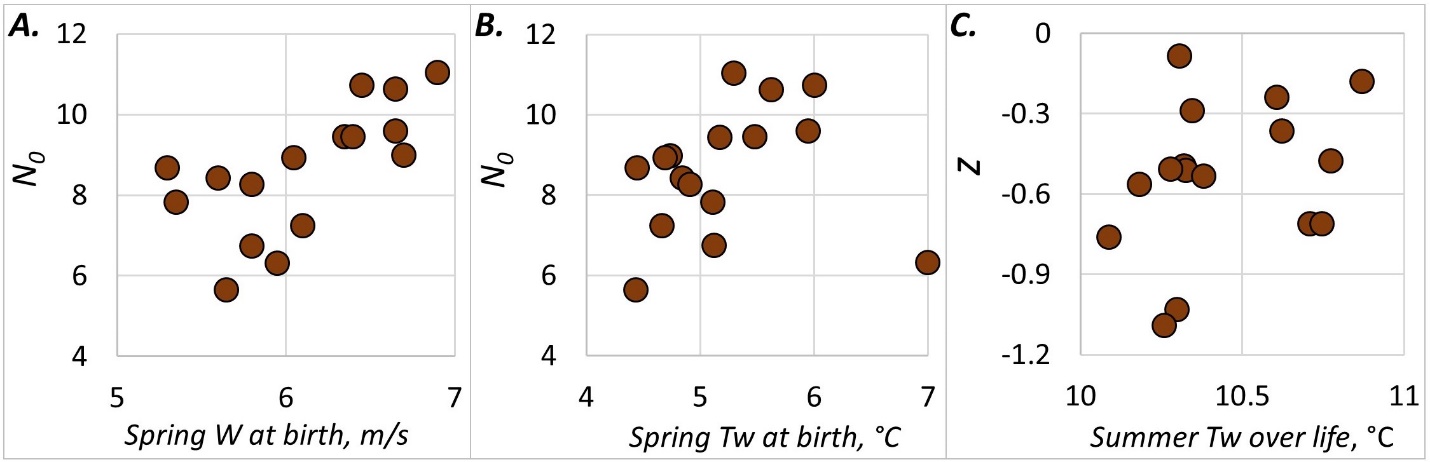
**

**Figure S3. The relationships between *N_0_*, *z* and selected environmental parameters.** **(A)** *N_0_* vs. wind speed in spring of the year of birth. **(B)** *N_0_* vs. surface water temperature in spring of the year of birth. **(C)** *z* vs. summer surface water temperature averaged over the lifespan.
